# Fibroblasts: a diverse population of cells balancing homeostasis, wound healing, regeneration, inflammation, fibrosis, and cancer across organs

**DOI:** 10.1172/jci.insight.202529

**Published:** 2026-07-08

**Authors:** Xiaochun Yang, Marcella Steffani, Sandeep Nadella, Dean Sheppard, Florian Rieder, Yuval Rinkevich, Rafael Kramann, David A. Tuveson, Robert F. Schwabe

**Affiliations:** 1 Department of Medicine, Columbia University, New York, New York, USA.; 2 Cold Spring Harbor Laboratory Cancer Center, Cold Spring Harbor, New York, USA.; 3 Northwell Health, New Hyde Park, New York, USA.; 4 Division of Pulmonary and Critical Care Medicine, UCSF, San Francisco, California, USA.; 5 Department of Inflammation and Immunity, Cleveland Clinic Research, and; 6 Department of Gastroenterology, Hepatology and Nutrition, Digestive Disease Institute, Cleveland Clinic, Cleveland, Ohio, USA.; 7 Chinese Institutes for Medical Research, Institute of Regenerative Biology and Medicine, Beijing, China.; 8 School of Basic Medical Sciences, Capital Medical University, Beijing, China.; 9 New Cornerstone Science Laboratory, Beijing, China.; 10 Department of Nephrology and Clinical Immunology, RWTH Aachen; Medical Faculty, Aachen, Germany.; 11 Department of Internal Medicine, Nephrology and Transplantation, Erasmus Medical Center, Rotterdam, Netherlands.; 12 Herbert Irving Comprehensive Cancer Center, New York, New York, USA.; 13 Columbia University Digestive and Liver Disease Research Center, New York, New York, USA.; 14 Institute of Human Nutrition, New York, New York, USA.

## Abstract

As the principal ECM-producing cell type, fibroblasts are essential regulators of tissue architecture and function in development, homeostasis, and disease. While their disease-promoting functions in fibrosis have long been the center of attention, it is increasingly recognized that fibroblasts exert critical homeostatic roles across organs, acting as sentinels that regulate the function, proliferation, and recruitment of epithelial, endothelial and immune cells in health and disease. Here, we will review the roles of fibroblasts and fibroblast-like cells in tissue maintenance, physiological wound healing, regeneration, maladaptive fibrosis, and cancer across major organs, including the skin, lung, liver, intestine, and kidney, and highlight organ-specific and shared populations and functions. We will discuss the role of PI16^+^ and COL15A1^+^ universal fibroblasts, organ-specific fibroblasts, and pericyte and pericyte-like stellate cells as cellular sources for the majority of CTHRC1^+^ activated fibroblasts and αSMA^+^ or LRRC15^+^ myofibroblasts and highlight the functions of specialized subpopulations, such as inflammatory fibroblasts, antigen-presenting fibroblasts, and fibroblast-like cells, including mesothelial and smooth muscle cells. A refined understanding of fibroblast heterogeneity holds promise for novel therapeutic concepts, aimed at targeting pathogenic subpopulations while preserving or enhancing homeostatic functions.

## Introduction

Fibroblasts are key drivers of fibrosis but are also found across organs and tissues in healthy states, where they contribute to organ architecture and homeostasis. While there has been a strong interest in understanding and targeting fibroblasts due to the pathogenic role of fibrosis, which may contribute to up to 45% of deaths globally (1, 2), the homeostatic functions of fibroblasts, often lost in progressive disease, are becoming increasingly recognized. Here, we will provide an in-depth review on critical balance between homeostatic and pathogenic functions of fibroblasts in health and disease, across organs. Our Review will highlight shared and organ-specific fibroblast populations and their functions. As epithelial cells, via the epithelial-mesenchymal transition, or bone marrow-derived fibrocytes do not represent major sources of extracellular matrix (ECM), in recent single cell-based analyses (3–13), this Review will focus selectively on fibroblasts and fibroblast-like cells as major source of ECM across organs.

### Fibrosis.

Defined as an excessive accumulation of ECM, fibrosis is a component of disease processes in nearly every organ, including the liver, lung, kidney, and gastrointestinal (GI) tract, as well as in a wide range of cancers. In acute injury, fibrosis exerts beneficial functions, promoting wound healing and regenerative responses. ECM, providing mechanical stability, and fibroblast-derived growth factors, promoting regeneration, act in concert to restore normal or near-normal tissue architecture. However, fibrogenic wound healing may become pathogenic if the underlying disease is not cured, leading to maladaptive and overshooting repair processes that result in excessive fibrosis and impaired organ function. Thus, disease duration is often a critical determinant of whether fibrogenesis is physiological or pathological. With a rise of chronic disease processes and an increase in life expectancy, pathological fibrosis is becoming more prevalent in modern societies.

Physiological fibrosis is best exemplified by acute injury of the skin, where through a well-coordinated process, fibroblasts are recruited to and proliferate at sites of injury, produce provisional ECM, contract for wound closure, and reorganize and strengthen tissue to achieve a stable scar (2, 14). Successful scar formation ensures mechanical stability and restoration of an intact barrier. Similar processes occur in the heart, where fibrosis following myocardial infarction provides mechanical stability, preventing rupture (15). Under some circumstances, wounds can heal without a scar, e.g., in fetal or mucosal wound healing or in some mammalian species such as spiny mice and reindeer (16–21). In other settings, fibrosis represents a temporary stage in the wound healing process, e.g., in highly regenerative tissues like the liver, where normal or near-normal tissue architecture can be achieved within weeks in mice and in months to years in humans, if injury and scar build-up have not been excessive (22).

Pathological fibrosis mostly occurs in the setting of chronic diseases and impaired regeneration. In such settings, continuous repair processes and fibrogenesis lead to the excessive accumulation of ECM, which negatively affects mechanical properties and contributes to the loss of healthy parenchyma and an altered milieu, which collectively impair organ function and contribute to disease progression. Pathological fibrosis is often associated with the failure to clear the underlying disease, which in turn leads to the chronic and maladaptive stimulation of fibrogenic processes. The consequences of fibrosis are often organ-specific, ranging from impaired gas exchange in the lung, altered contractility or motility, to altered blood flow, and result in diminished organ function (15, 23–27). Evolutionary pressure may have favored the benefits of acute wound healing over the complications of chronic wound healing, as acute wound healing ensures survival and reproductive fitness. The negative effects of chronic wound healing often manifest in older age and do not significantly affect the reproductive fitness of populations. Furthermore, progressive fibrosis in the liver, kidney, or cardiovascular system is often caused by “modern” chronic diseases that were rare during mammalian evolution, such as hypertension, metabolic syndrome, or chronic infections with viruses such as hepatitis B and C (28).

### Fibroblasts.

On a cellular level, fibrosis is primarily mediated by mesenchymal cells, commonly referred to as fibroblasts. First referred as “spindle cells” by Virchow (29), the term fibroblast was later coined by Ziegler (30), describing ECM-producing cells with a prototypical shape. The most classifications distinguish between fibroblasts and myofibroblasts, which express α-smooth muscle actin (αSMA) and have contractile properties for wound contraction and closure (31–33). Lipid droplet-containing fibroblasts, such as lipofibroblasts in the lung (34), or hepatic stellate cells (HSCs) in the liver (28, 35) represent additional morphologically distinct populations. Recent studies, applying single-cell RNA-seq (scRNA-seq) across tissues and disease states, have uncovered a much broader degree of diversity with up to 20 distinct subtypes, often classified by specific markers or gene expression patterns as well as functional categories, such as myofibroblasts and inflammatory, remodeling, progenitor-like and antigen-presenting fibroblasts (3, 4, 7, 36). Furthermore, mesenchymal fibroblast-like cells such as pericytes (which include stellate cells), smooth muscle cells (SMCs), and mesothelial cells may represent fibroblast precursors, which contribute to fibrosis in some organs or specific settings (4, 37–41).

## Fibroblast and fibroblast-like subpopulations

Fibroblasts exist in every organ, where they maintain tissue architecture, support the function of other cell types, and promote regeneration and repair in case of injury (2, 42). While there are many organ-specific features, recent transcriptomic studies have also highlighted common fibroblast features and subtypes across organs (2, 42). Organ-specific differences appear more pronounced in homeostasis, where fibroblasts are adapted to organ-specific environments, than in fibrosis, where activated fibroblasts reach similar end states with high activation. For example, in the lung, fibroblasts secrete elastin to provide elastic properties needed for breathing (43), support the production of surfactant to reduce surface tension in alveoli, and provide a supportive niche for the maintenance of alveolar type 2 (AT2) cells (44, 45). The liver contains a pericyte-like cell population, termed hepatic stellate cells, which store the majority of the body’s vitamin A and regulate liver size as well as the metabolic and detoxifying functions of hepatocytes (11, 28, 40, 46, 47). In the kidney, a fibroblast-like cell type is responsible for oxygen sensing and the production of erythropoietin (48). Furthermore, each organ has a different regenerative capacity, with the liver representing one of the most regenerative and the heart one of the least regenerative organs (49, 50). Therefore, fibroblasts have essential functions in liver regeneration (11, 28), whereas such functions have not been described in low-regenerative organs, such as the heart.

### Fibroblasts heterogeneity across organs in health and disease.

scRNA-seq–based studies have provided deep insights into fibroblast heterogeneity and allow for the comparison of fibroblast heterogeneity within specific organs and across organs. While there is no universally accepted global classification of fibroblast subpopulations and the number and markers of reported subpopulations vary between studies, there are several conserved fibroblast subpopulations with common markers and/or functions (Table 1). For detailed lists of all recently uncovered specialized subpopulations, we refer to mouse and human studies that have analyzed fibroblast heterogeneity in large multiorgan datasets, including Buechler et al. (230,000 fibroblasts across 17 tissues, 50 datasets, 11 disease states, and 2 species), who described 10 fibroblast clusters in homeostasis and 10 fibroblast clusters in perturbed states in mice and 6 fibroblasts clusters in perturbed states from patients (3); Gao et al. (269,899 cells from 517 human samples, 11 tissues, and 43 healthy and diseased states), who described 20 fibroblast clusters (4); and Liu et al. (249,156 fibroblasts from 73 studies across 10 human tissues), who uncovered 18 fibroblast clusters (7). As there are considerable challenges in generating single-cell suspensions from highly fibrotic tissues, fibroblasts may be under-, over-, or misrepresented in isolation-based studies owing to difficulties of retrieving fibroblasts deeply embedded into ECM or marker-based isolation not representing all fibroblasts equally. Furthermore, integrating data from different organs and studies may create batch effects despite substantial advances in this area. Finally, various definitions have led to the exclusion of populations, such as pericytes or SMCs from some analyses. Despite these limitations, one consistent finding emerging from these multiorgan analyses is the presence of subpopulations shared across organs. Utilization of single-nucleus RNA-seq (snRNA-seq) may provide a less biased approach to study fibroblasts across organs but was not utilized for the above-discussed large multiorgan datasets.

**Figure 1:**
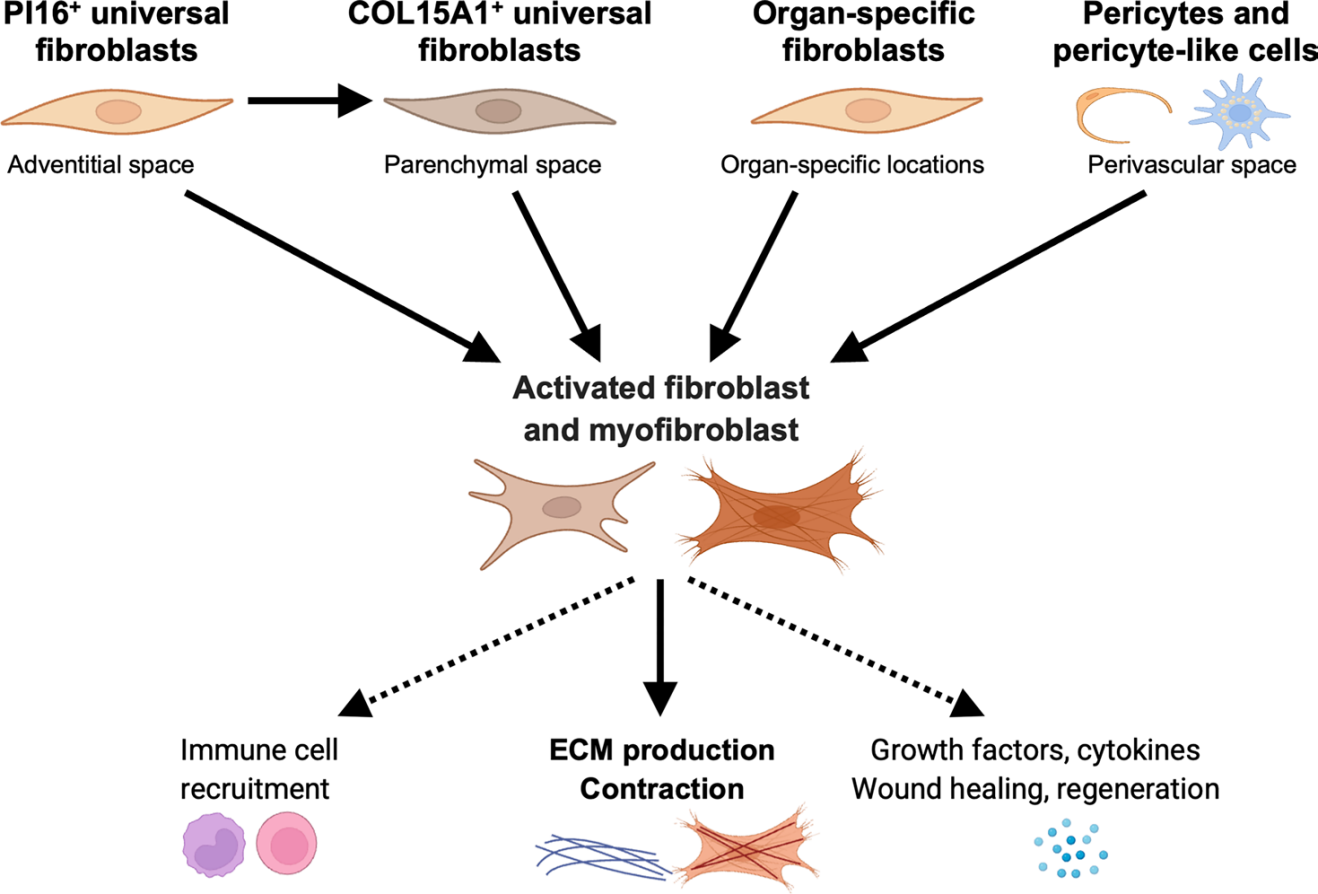
Main cellular sources of ECM-producing fibroblasts and myofibroblasts. PI16^+^ and COL15A1^+^ universal fibroblasts, organ-specific fibroblasts, and pericytes and pericyte-like cells represent the major sources of fibroblasts across organs, contributing to ECM production, wound contraction, inflammatory cell recruitment, regeneration, and wound healing. With the exception of the liver, pericytes/pericyte-like cells likely only make minor contributions to ECM-producing activated fibroblasts.

Universal fibroblasts, also termed fibroblast progenitors, exist as two different subtypes, namely PI16^+^ fibroblasts and COL15A1^+^ fibroblasts (3) (Figure 1). PI16^+^ fibroblasts represent a fibroblast subpopulation that has been found consistently across organs and studies (3, 4, 7). PI16 is a peptidase inhibitor that has been implicated in the regulation of inflammation (51). PI16^+^ fibroblasts were found in the lung, skin, stomach, colon, pancreas, kidney, breast, and synovium and express additional markers such as dipeptidyl peptidase 4 (DPP4), DPT, CD34, and LY6C1 (3, 4, 7) (Table 1). PI16^+^ fibroblasts are concentrated close to the vasculature in most tissues and are often described as adventitial fibroblasts though they can also be found away from the vasculature in some contexts (51). Based on studies in which PI16 was knocked out or in which PI16^+^ fibroblasts were cocultured with immune cells (52), it has been suggested that PI16^+^ fibroblasts contribute to the extravasation and infiltration of immune cells (51), a feature that is considered a crucial driver of fibrosis across organs and diseases (53, 54). COL15A1^+^ fibroblasts constitute a second group of universal or progenitor fibroblasts that are found across organs (3, 4, 7). Collagen XV is associated with the basement membrane zone in most tissues. Accordingly, gene expression analyses suggest that COL15A1^+^ fibroblasts primarily produce basement membrane components, such as COL4A1, HSPG, LAMA2, and COL18A1, and mediators with a role in regeneration and proliferation, such as APOD, APOE, IGF1, and MDK (3, 4).

Matching this gene expression, PI16^+^ fibroblasts are primarily located in the adventitial space, whereas COL15A1^+^ fibroblasts are associated with parenchymal regions (3, 4, 55). Interestingly, collagen XV has been shown to be tumor-suppressive (56), further confirming the homeostatic and epithelial-supporting role of COL15A1^+^ fibroblasts. In summary, their location and gene expression suggest COL15A1^+^ fibroblasts as a homeostatic cell population that maintains epithelial health and regeneration, contrasting the vascular-associated functions of PI16^+^ fibroblasts (4). Trajectory studies suggest that PI16^+^ fibroblasts may represent the source of many other fibroblast populations across organs, including COL15A1^+^ fibroblasts (3). Both the PI16^+^ and COL15A1^+^ universal fibroblast populations can transdifferentiate further into pathogenic and specialized fibroblast subpopulations, including highly activated CTHRC1^+^ fibroblasts and LRRC15^+^ myofibroblasts (3, 4) (Figure 1). It is possible that the PI16^+^ universal fibroblast population is similar to perivascular fibroblasts labeled by Gli1 (57). However, the role of these two universal populations as precursors of activated fibroblasts and fibroblasts requires further confirmation in most organs.

### Organ-specific fibroblasts.

Although recent scRNA-seq–based atlases have suggested the presence of universal fibroblast populations across organs, the aforementioned limitations in these studies (tissue digestion, pooling of scRNA-seq data from different studies, focus on cancer and lack of studies where fibroblasts from various organs come from the same donor) may have overshadowed organ-specific fibroblast populations and features in health or disease. Examples of fibroblasts with organ-specific functions include alveolar fibroblasts, which support surfactant production (44, 45) as well as erythropoietin-producing Norn cells in the kidney (48) (Figure 1 and Table 1).

Pericytes or pericyte-like cells, which wrap around capillaries and maintain vascular functions, represent another key population that can give rise to ECM-producing fibroblasts in addition to their blood vessel-dependent functions (41) (Figure 1). While Buechler et al. excluded pericytes from their analyses, Gao et al. reported a large RGS5^+^, NOTCH3^+^, CD36^+^ cluster that — in addition to the PI16^+^ and COL15A1^+^ universal fibroblast clusters — could give rise to protomyofibroblast and LRRC15^+^ myofibroblast clusters in trajectory analyses in their multiorgan dataset (4). Likewise, Liu et al. reported an RGS5^+^ fibroblast population that expresses PDGFRB, COL4A1, and MYL9 and was found in every investigated organ, including breast, lung, pancreas, liver, colorectum, ovary, stomach, prostate, kidney, and head and neck to varying degrees (7). This finding is consistent with sc/snRNA-seq–based and lineage tracing studies that have demonstrated a key contribution of pericytes as a source of fibroblasts and myofibroblasts in the liver, kidney, lung, and heart (41), yet with different relative contributions (Figure 1). While several pericyte markers exist, such as NG2 and PDGFRβ, these are not specific to pericytes and expressed by other mesenchymal cell-types such as vascular smooth muscle. Likewise, Gli1 does not specifically mark pericytes, but perivascular fibroblasts (57), which are key contributors to fibrosis and their detachment might explain the loss of vasculature after fibrosis triggering hypoxic organ injury (58, 59). In the liver and pancreas, stellate cells represent a pericyte-like cell population that stores retinyl esters and exerts key roles in liver and pancreas fibrosis (28, 60). However, as stellate cells are mostly NG2^–^, they may also represent an organ-specific perivascular fibroblast population rather than a pericyte-like cell type. In other organs, pericytes represent a variable and potentially minor source of fibroblasts with a primary role in maintaining vascular integrity, as their contribution to kidney, heart, and lung fibrosis may have been overestimated (6, 41, 61–67).

Activated fibroblasts and myofibroblasts comprise a group of fibroblast subpopulations that are highly activated and associated with perturbed and cancer states. Fibroblasts that are markedly fibrogenic without expressing αSMA are often referred to as activated or profibrotic fibroblasts and have been identified by the expression of CTHRC1 in scRNA-seq studies in multiple organs, including the lung, heart, and liver (12, 68–70) (Table 1). Myofibroblasts are often considered the terminal stage of fibroblast differentiation and are characterized by their contractile properties and expression of αSMA or leucine-rich repeat containing 15 (LRRC15) in addition to fibrogenic properties (3, 4, 31, 33, 71) (Table 1). However, current multiorgan atlases do not distinguish between CTHRC1^+^ fibroblasts and myofibroblasts, and one type of fibroblast does not necessarily express higher levels of ECM than the other. LRRC15^+^ fibroblasts may derive from PI16^+^ and COL15A1^+^ universal fibroblasts and from pericytes (3, 4) (Figure 1). While some studies described LRRC15^+^ myofibroblasts in arthritis, skin wounding, and pancreatic cancer and to a lesser degree in lung, liver, and high-fat diet–induced arterial injury (3, 72), functional characterization of LRRC15^+^ myofibroblasts has been primarily performed in the context of cancer (71, 73). The development of LRRC15^+^ myofibroblasts from dermatopontin^+^ universal fibroblasts requires TGF-β receptor type 2 signaling, as demonstrated by in vivo genetic approaches (73). The classification and function of other myofibroblast subpopulations are less clear. Guo et al. found that MMP1^+^ fibroblasts, HOPX^+^ fibroblasts, and SFRP2^+^ fibroblasts showed intermediate states between terminally differentiated LRRC15^+^ myofibroblasts and myofibroblast precursors and described them therefore as protomyofibroblasts. MMP1^+^ fibroblasts exhibited inflammatory features and were also described as inflammatory myofibroblasts in another study that integrated skin fibroblast subtypes with fibroblasts from other organs, expressing high levels of IL11, CXCL8, and IL7R (36). However, the HOPX^+^ fibroblasts and SFRP2^+^ fibroblast subtypes have not been described in other multiorgan fibroblast atlases.

Inflammatory fibroblasts are found in the immune system; barrier organs such as skin, lung, and GI tract; and in the context of chronic inflammation, fibrosis, and cancer (3, 4, 7, 12, 36, 74–80) and exist as different subpopulations. CCL19^+^CCL21^+^ fibroblastic reticular cells (FRCs), described in the multiorgan atlas from Buechler et al. (3), are well-characterized specialized immunoregulatory mesenchymal cell types, typically found in lymph nodes and the spleen, where they regulate T and B cell responses (75, 81, 82), as well as in the skin, and intestine, where they enhance immunity in these critical barrier organs and regulate innate lymphoid cells (36, 76). In the lung, adventitial fibroblasts may fulfil a similar role in regulating type 2 innate lymphoid cells via IL33 (77). Gao et al. describe three distinct subsets of inflammatory fibroblasts, including IL6^+^ fibroblasts, HGF^+^ fibroblasts, and HSPA6^+^ fibroblasts (4). Other multiorgan atlases and studies described IL6^+^ fibroblasts across tissues, including breast, lung, pancreas, liver, colorectum, ovary, stomach, prostate, kidney, synovium, and head and neck but to varying degrees (7, 80). The secretion of inflammatory mediators such as IL6 can be increased in fibroblasts from virtually any organ by treatment with TNF and IL1 (83). Functionally distinct subsets of CXCL10^+^CCL19^+^ immune-interacting and SPARC^+^COL3A1^+^ vascular-interacting fibroblasts were described in chronic inflammatory diseases, such as rheumatoid arthritis, inflammatory bowel disease (IBD), interstitial lung disease, and Sjögren’s syndrome, in patients and mouse models (79). Pseudotime analysis has suggested inflammatory fibroblasts as precursors of fibrotic fibroblasts (12). Collectively, these findings position inflammatory fibroblasts as critical effectors of tissue pathology, with both shared and organ-specific features.

Antigen-presenting fibroblasts are found across organs as well as in tumors in scRNA-seq–based studies and multiorgan fibroblast atlases (4, 7, 12, 84–87). CD74 is the most commonly used marker for antigen-presenting fibroblasts (4, 7). Expression of MHC class II represents another characteristic feature, related to their function as antigen-presenting cells (88). Interferon **γ** is a key driver of the switch toward an antigen-presenting fibroblast phenotype (86, 89). Moreover, IL1 and TGF-β have been described to induce antigen-presenting cancer-associated fibroblasts (CAFs) in the setting of cancer (85). Antigen-presenting fibroblasts can be derived from different sources, including fibroblasts and mesothelial cells (86, 88). Besides antigen presentation and stimulation of immune responses, which requires the presence of costimulatory molecules such as CD40, CD80 and CD86 (present on some but not all antigen-presenting CAFs), antigen-presenting fibroblasts may also increase T cell differentiation toward a Treg phenotype (85). Whether antigen-presenting fibroblasts fulfill protective or disease-promoting functions, as seen for example in cardiac pressure overload (86), requires further investigations and will likely reveal disease- and organ-specific roles.

### Fibroblast-like cells.

Besides, fibroblasts and their precursors, such as pericytes, SMCs, and mesothelial cells, exist in most organs and are often described as fibroblast-like cells that can perform fibroblast-like functions in specific anatomic niches and settings.

SMCs are specialized contractile cells, whose primary function is the regulation of blood vessel diameter and blood pressure in arteries and veins, the regulation of bronchial diameter in the respiratory system, and peristalsis in the GI tract (90, 91). MYH11 is the most common marker for SMCs in scRNA-seq studies and multiorgan fibroblast atlases (3, 4). However, MYH11 can also be expressed by pericytes, e.g., in the heart (67). scRNA-seq has revealed transcriptional heterogeneity among SMCs in arteries from different organs or locations but a more conserved pattern in venous SMCs (91). Characterized by the expression of contractile proteins such as αSMA, SMCs exhibit remarkable phenotypic plasticity and express genes commonly found in fibroblasts and pericytes, including ECM such as collagens and filaments such as desmin and transgelin (91). Accordingly, SMCs may contribute to pathological processes such as atherosclerosis, where they proliferate, migrate, and synthesize extracellular matrix to form fibrous caps (92), or airway remodeling and asthma in the lung (91, 93). Together, these findings highlight SMCs as a specialized and plastic cell type that may regulate vascular tone and fibrosis but is often not included in multiorgan fibroblast analyses.

Mesothelial cells exhibit both epithelial and mesenchymal features and can undergo a process termed mesothelial-mesenchymal transition to contribute to the fibroblast pool and fibrogenesis. Mesothelial cells form a monolayer, known as the mesothelium, lining the peritoneal, pleural, and pericardial organs and cavities (94). MSLN and UPK3B are common markers for mesothelial cells in scRNA-seq analyses and multiorgan atlases (3, 4). Interestingly, mesothelial cells cluster closely to COL15A1^+^ fibroblasts and inflammatory fibroblasts, highlighting their transcriptional similarity to fibroblasts (4). Multiorgan analysis has revealed two mesothelial subpopulations in homeostasis: a Tmsb10^+^ population found across organs and an ACTG1^+^ population restricted to abdominal organs (95). In diseased states, “metabolically active,” “fibrogenically active,” “proteolytically active,” and “immune-modulatory” subpopulations can be found (95). These findings are consistent with a role of mesothelial cells not only in capsular biology, but also subcapsular fibrosis, as demonstrated by subcapsular mesothelial cell–derived myofibroblasts in liver and lung (95–97).

## Fibroblasts in homeostasis and regeneration

**Figure 2:**
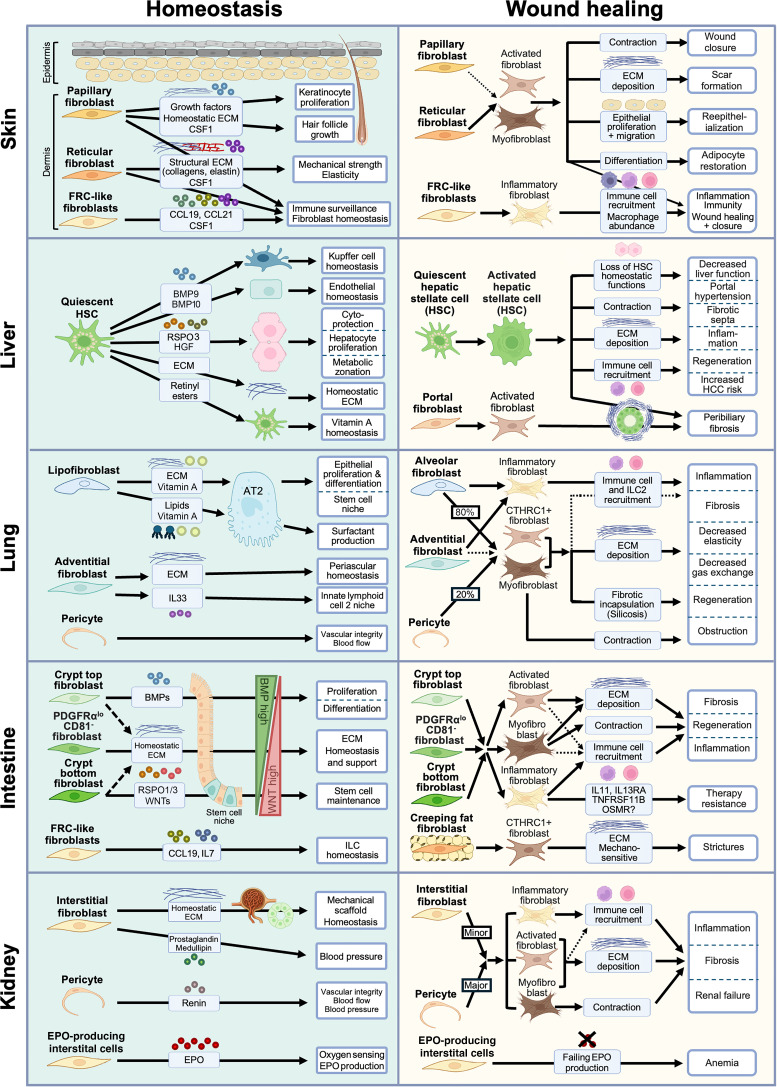
Homeostatic and injury-associated functions of fibroblasts across organs. Displayed are the main fibroblast subpopulations and their roles in homeostasis and wound healing across five major organs.

Fibroblasts exert critical homeostatic functions, providing a matrix that is crucial for organ structure and function by producing a wide range of ECM components, including fibrillar and nonfibrillar collagens, hyaluronic acid, elastin, laminins, nidogen, perlecan, and fibronectin (98). In addition to fibroblast-produced ECM providing pure structural support and organ-specific mechanical properties, fibroblasts also actively crosstalk with other cells, such as epithelia and immune cells, through signaling pathways that control their differentiation state, migration, turnover, and regeneration (4, 45, 82) (Figure 2). While many of these homeostatic and proregenerative signaling pathways are activated by fibroblast-derived growth factors, such as WNTs and R-spondins in epithelia, fibroblast growth factors, and hepatocyte growth factor (49), ECM may also control epithelial gene expression and proper functions through ECM receptors and mechanosensing (99–101). These critical regulatory functions of fibroblasts are further highlighted by their role in development, where fibroblasts provide important positional information for other cell types as well as cues for growth and differentiation (45, 102, 103), including collagen type XV, produced by COL15A1^+^ universal fibroblasts (56), highlighting the fibroblast-derived cues as gatekeepers of epithelial cell differentiation. The pathways by which fibroblasts control epithelial cell proliferation and turnover during development and homeostasis are often activated after injury, mediating tissue regeneration. Proregenerative capacities of fibroblasts are observed in organs with both higher and lower regenerative capacity, including liver, intestine, skin, and lung (49). In summary, a primary function of fibroblasts across organs lies in the maintenance of epithelial health in homeostasis and its restoration after loss of epithelial cells (Figure 2). Furthermore, fibroblasts are important components of multicellular niches that support organ-specific functions, such as the immune barriers in organs that share surfaces with the environment, such as the skin, intestine, and lung (36, 76–78), as well as organ function, such as the production of surfactant in the lung (44, 45) and the regulation of metabolic functions in the liver (11) (Figure 2).

### Skin.

Dermal fibroblasts provide structural strength and flexibility to the skin via multiple ECM components, including collagens, elastin, hyaluronic acid, proteoglycans, and glycosaminoglycans (104). The ECM structural organization in the skin varies to balance between tensile strength and elasticity in specific regions of the body. Fibroblast subtypes are adapted to specific function with the distinct anatomical layers of the skin, including upper papillary dermis, the lower reticular dermis, hypodermis, and fascia, which contain adipocytes and preadipocytes (Figure 2). A recent study, combining single-cell approaches and spatial transcriptomics, characterized six major fibroblast subtypes in human skin (36). Two fibroblast types, PI16^+^ universal (reticular) and superficial (papillary) fibroblasts, are located throughout the skin at different depths (36). The remaining fibroblasts were specialized in function and location, including follicular reticular cell–like CCL19^+^ fibroblasts, which maintain immune functions; perivascular fibroblasts; ASPN^+^COL11A1^+^ hair follicle fibroblasts; and Schwann-like fibroblasts (36). The large number of fibroblast populations, which can be further subclustered into even more specialized subpopulations (36), reflect the diverse functions of the skin in maintaining mechanical properties, an immune barrier, supporting hair and nerve-glandular functions in skin development and homeostasis (105). In mice, a DPP4/CD26-expressing fibroblast subtype, lineage-labeled by Engrailed1-Cre, accumulated postnatally and migrated to the reticular dermis, accounting for about 75% of skin fibroblasts at days P30 and P56 and likely representing the mouse equivalent of PI16^+^ universal fibroblasts (106). Adult skin contains a fibroblast progenitor in its deepest connective tissue fascia layers, marked by protein C receptor (Procr) and PI16, which generates the majority of inflammatory and myofibroblasts in deep skin wounds (14, 107). Distinct functions of fibroblast subtypes were observed in mice, where transplantation of mixed dermal cell suspensions lacking the upper dermis papillary fibroblasts could not regenerate papillary dermal ECM or hair follicles (108). The proregenerative capacity of papillary and reticular fibroblasts differs as papillary Lef1^+^ fibroblasts more effectively support regeneration and wound healing than reticular fibroblasts (109, 110). Following injury and wound healing, myofibroblasts may differentiate into adipocytes, an important cell type of the healthy skin, under the influence of BMP signaling, highlighting a restorative function of fibroblasts (111). Dpt^+^ skin fibroblasts also secrete CSF1 to maintain CD64^+^ and CD11c^+^ macrophages, which in turn regulate fibroblast numbers, metabolism, and immune signaling, rendering this bidirectional fibroblast-macrophage interplay critical for skin integrity in homeostasis and disease (Figure 2) (78). Fibroblasts also contribute to the loss of homeostatic skin functions during aging, as senescent fibroblasts can induce and accelerate age-related dysfunction of other skin cells (112). In summary, multiple fibroblast populations support the skin and its barrier functions, maintaining integrity, immune defenses, and specialized functions related to hair growth and nerve functions (Figure 2).

### Liver.

HSCs constitute the main mesenchymal cell population and are often considered a liver-specific pericyte, located in the perisinusoidal space of Disse (28, 35). Although HSCs are best known as primary fibrogenic cells that mediate fibrosis and contribute to portal hypertension (28, 40, 46), there is increasing understanding of the role of quiescent HSCs in liver homeostasis (11, 28) (Figure 2). One primary function of quiescent HSCs is the storage of vitamin A in the form of retinyl esters in their characteristic lipid droplets (47). Furthermore, quiescent HSCs regulate many functions of the healthy and regenerating liver (Figure 2). Livers lacking HSCs or HSC-expressed R-spondin 3 (RSPO3) are smaller, suggesting a failure of homeostatic liver regeneration (11, 113). Similarly, liver regeneration after 70% partial hepatectomy is strongly reduced in mice with HSC depletion or HSC-specific knockout of *Rspo3* (11). Finally, several hepatocyte functions, including metabolic zonation and detoxification, are also regulated via HSC-expressed RSPO3 (11, 28, 113). Quiescent HSCs also express high levels of hepatocyte growth factor, which protects the liver from injury (114), as well as BMP9 and BMP10, which maintain Kupffer cell and endothelial cell identity and functions (115). Together, these data suggest that HSCs are central regulators of the multicellular network that maintains liver homeostasis and functions. Additional stromal cell populations in the liver include portal fibroblasts, which surround portal triads and likely contribute to homeostatic matrix and functions in this important anatomic niche (116), as well as vascular SMCs (Figure 2).

### Lung.

Fibroblast-produced ECM is critical for its elasticity, which allows expansion during inhalation and recoil during exhalation, thereby ensuring efficient gas exchange (43). scRNA-seq–based studies have revealed four major stromal cell populations in homeostatic conditions in addition to airway SMCs and vascular SMCs in mice and humans (12, 117). (a) Alveolar fibroblasts (often also referred to as lipofibroblasts owing to high lipid droplets and lipid-processing genes in mice, less in humans), characterized by the expression of Col13a1, Itga8, Inmt, Ces/Ces1d and Scube2 (in mice but not humans), reside in a distinct anatomic niche close to AT2 cells (117). Besides maintaining a supportive niche for their growth and differentiation into AT1 cells via FGF10-FGFR, alveolar fibroblasts support AT2 cells in the production of surfactant (44, 45) (Figure 2). Accordingly, depletion of alveolar fibroblasts reduced the number of AT2 cells by 30%–40% in homeostasis (12). (b) Adventitial fibroblasts are PI16^+^, resembling PI16^+^ universal fibroblasts in other organs. They are located in interstitial spaces surrounding bronchovascular bundles and provide structural support for this critical anatomic niche (117). Furthermore, adventitial fibroblast subsets express IL33 and support a niche for immune cells (77) (Figure 2). (c) Peribronchial fibroblasts are LGR5^+^ cells located beneath airway epithelial cells and intercalated with airway SMCs. They likely contribute to bronchial anatomy and functions, but their role in homeostasis requires further studies (117). (d) Ductal myofibroblasts wrap alveolar ducts in a fashion similar to how airway SMCs wrap larger conducting airways and share the expression of LGR6 with airway SMCs, suggesting similar function. Although their role in the developing lung and secondary septum formation is established, the homeostatic roles of ductal myofibroblasts persisting in the adult lung require further investigation (117). During aging, lung mesenchymal stromal cells lose their ability to support the growth of AT2 cells and their capacity to revert to a proregenerative state following bleomycin-induced activation (118, 119).

### Intestine.

Intestinal fibroblasts are found in different layers of the intestine and exert a central role in maintaining WNT and BMP gradients that keep WNT signals high in the crypt and low in the villus and, vice versa, BMPs high in the villus but low in the crypt (120, 121) (Figure 2). Accordingly, crypt-bottom fibroblasts or trophocytes, marked by the expression of CD81 and low expression of PDGFRα, are the primary cellular source of WNTs, such as *Wnt2* and *Wnt2b*, the BMP antagonists Gremlin 1 and 2 and WNT signaling mediators RSPO1 and RSPO3 (10, 13, 120) (Figure 2). These cells resemble DPT^+^ PI16^–^ universal fibroblasts in other organs (3). PDGFRα^hi^ fibroblasts, also referred to as telocytes or crypt-top fibroblasts, are characterized by high expression of BMP2, BMP4, BMP5, BMP7, and WNT antagonist DKK3. An additional population of surrounding stroma is PDGFRα^low^ and CD81^–^ (120, 121). The crypt-bottom PDGFRα^low^ population of telocytes has a key role in supporting intestinal stem cell proliferation (121) (Figure 2). Accordingly, deletion of RSPO3 in crypt fibroblasts, either alone or alongside deletion of RSPO3 in lymphatic endothelial cells, reduced the number of intestinal stem cells, villi length, and repair after injury (122, 123). GREM1^+^RSPO3^+^ crypt-bottom fibroblasts collaborate with RSPO3^+^ lymphatic endothelial cells to maintain intestinal stem cells and epithelial health (124). scRNA-seq studies of the small intestine and colon show a similar but more refined picture, showing additional populations of myofibroblasts and SMCs, expressing high levels of αSMA and MYH11, as well as a cluster showing features of antigen-presenting fibroblasts with high expression of CD74 (5, 125, 126). Similar to other barrier organs, such as the skin and lung, the intestine also contains FRC-like cells, which control intestinal lymphoid cell homeostasis and immune regulation via the secretion of cytokines such as IL7 (76) (Figure 2). Together, these studies highlight the critical role of distinct fibroblast populations in establishing a signaling gradient along the small intestinal villus–crypt and colonic crypt top–bottom axes, thereby supporting intestinal stem cells maintenance and differentiation (121, 127) as well as in antigen-presentation and immunity at this critical host-microbiome barrier (Figure 2).

### Kidney.

The kidney contains several mesenchymal cell types, including fibroblasts, pericytes, mesangial cells, and vascular SMCs. While these populations share a mesenchymal origin, they show distinct transcriptional signatures in scRNA-seq analyses and functional specialization (128). Both fibroblasts and pericytes express PDGFRβ and CD73, consistent with a shared progenitor origin (129). Interstitial fibroblasts and pericytes contribute to renal structural integrity by producing homeostatic extracellular matrix (6, 63) (Figure 2). Renal erythropoietin-producing cells, recently also referred to as Norn cells, are a specialized subpopulation of Cxcl14^+^, Col1a1^+^, Dcn^+^, Lpar1^+^ fibroblast-like interstitial cells in the renal cortex and outer medulla that, despite sharing a wide range of genes with pericytes and fibroblasts, cluster separately in scRNA-seq analyses (48). They link local oxygen sensing to erythropoietin production and systemic erythropoiesis (48) (Figure 2). Additional mediators produced by interstitial fibroblasts or fibroblast-like cells that regulate renal blood flow and blood pressure include renin, produced by cortical specialized pericytes (renin lineage cells), Cox2, and a not fully characterized mediator termed medullipin, produced by renal medullary interstitial cells (48, 130, 131) (Figure 2). Although several scRNA-seq atlases of healthy kidney exist, they have not analyzed the mesenchymal diversity in renal homeostasis in further detail (6, 132, 133). Unlike the liver and lung, the kidney has limited regenerative potential. Therefore, renal fibroblasts may regulate recovery from stress and injury but not proper regeneration. It has been proposed that pericytes serve as a local stem cell population that replenishes differentiated interstitial and vascular cells lost during aging (134). In summary, diverse populations of renal fibroblasts contribute to kidney and systemic homeostasis, including regulation of erythropoiesis and blood pressure (Figure 2).

## Fibroblasts in the development of organ fibrosis

Fibrosis is the consequence of maladaptive wound healing in the setting of chronic inflammation and injury due to unresolved chronic disease processes. Fibrosis is characterized by an excessive accumulation of ECM within and around inflamed or damaged tissues. This pathological remodeling disrupts normal physiological organ function and can ultimately lead to organ failure (135). In fibrosis, the equilibrium between ECM synthesis and degradation is lost with a greater increase in ECM deposition than ECM degradation, causing ECM accumulation and fibrosis (136). Moreover, upregulation of MMP inhibitors, such as TIMPs, disrupts proteolytic ECM turnover and shifts the balance toward ECM deposition and fibrosis (137). Similar to fibroblasts in the homeostatic state, fibroblasts in the setting of chronic disease and fibrogenesis show both shared and organ-specific gene expression profiles and subpopulations, with the upregulation of fibrillar collagens and contractile filaments representing shared features of activated fibroblasts and LRRC15^+^ myofibroblasts among organs (3, 4). Recent single-cell and fate tracing studies have suggested that a majority of activated fibroblasts arise from COL15A1^+^ universal fibroblasts, PI16^+^ universal fibroblasts, and, in the liver and pancreas, from pericyte-like stellate cells. However, it is possible that specialized and organ-specific fibroblasts may represent additional sources of activated fibroblasts and myofibroblasts (Figure 1). Furthermore, mesothelial cells (96, 138, 139) and SMCs (140, 141) contribute to localized fibrosis in specific anatomic locations, such as the organ capsule or subcapsular spaces and perivascular spaces, respectively. Beyond these specialized fibroblast-like populations, the localization of the underlying injury may ultimately determine which types of fibroblasts contribute to fibrosis. Examples of different patterns of fibrosis include pericentral and periportal fibrosis in the liver, and alveolar, perivascular, and peribronchial fibrosis in the lung.

### Skin fibrosis.

Dermal fibrosis can arise from any type of skin injury, including thermal burns, trauma, infection, radiation, surgery, or systemic diseases such as scleroderma (142). Multiple dermal cell populations — including fibroblasts, perivascular cells, and adipocyte progenitors (but not mature adult adipocytes) — may serve as sources of activated ECM-producing fibroblasts and myofibroblasts in injured or diseased skin (142, 143) (Figure 2). Lineage tracing, flow cytometry, scRNA-seq, and transplantation studies have revealed that CD26^+^ (also known as DPP4) and PI16^+^ universal-like fibroblasts have emerged as the dermal fibroblast lineage responsible for the majority of wound-induced ECM deposition in mice and patients in different types of skin injury (106, 144, 145). Anatomically, Procr^+^ and adult PI16^+^ fibroblast progenitors in the reticular dermis and subcutaneous fascial layers are the primary mediators of skin fibrosis (14, 107, 108), which is reflected by the finding that deeper skin wounds lead to stronger scarring of the skin (142, 146). These fibroblasts also contribute to the development of keloids, a form of hypertrophic skin scars (145). In systemic sclerosis, fibroblasts differentiate from an SFRP2^hi^DPP4-expressing progenitor fibroblast population into myofibroblasts through a two-step process, with a first step resulting in a global upregulation of transcriptome markers, such as PRSS23 and THBS1, followed by a transition into SFRP4- and FNDC1-expressing myofibroblasts with strongly upregulated TGF-β target genes (147, 148). A recent study described that Cyp26b1, a retinoid-metabolizing P450 cytochrome, was expressed by fibroblasts that were highly fibrogenic and at a terminal point in their trajectories and that Cyp26a1/b1 inhibitor talarozole ameliorated the development of bleomycin-induced skin fibrosis (149). Thus, the development of skin fibrosis following different types of injury appears to converge on CD26/DPP4-expressing fibroblasts, which are prone to activation into myofibroblasts and can be potentially inhibited by interference with retinoid signaling (142).

### Liver fibrosis.

The development of liver fibrosis, and its advanced form, cirrhosis, are the strongest predictors of clinical outcomes across multiple etiologies of end-stage liver disease (150, 151). HSCs are widely considered the primary fibrogenic cell type of the liver and cell of origin for activated fibroblast and myofibroblasts in toxic, metabolic, and biliary injury (28, 40, 152). HSCs undergo a well-characterized activation process that transforms them from homeostatic, vitamin A–storing pericyte-like cells into ECM-producing fibroblasts and myofibroblasts (23, 152) (Figure 2). Besides HSC activation by TGF-β, angiotensin, leptin, CTGF, and DAMPs from dying hepatocytes (153), HSC proliferation, mediated largely by PDGF, is also a key contributor to liver fibrosis, as demonstrated in mice with HSC-selective deletion of cyclin E1 (154). Portal fibroblasts make additional contributions to fibrosis in the periportal region, but the extent of this contribution remains controversial (28, 40, 116, 155) (Figure 2). Activated fibroblasts in the liver show significant heterogeneity, as noted both in mice with reporters for Col1a1 and αSMA (156), as well as in subsequent scRNA-seq studies, which demonstrated quiescent HSCs, activated HSCs, intermediate activated HSCs, reverted HSCs, inflammatory HSCs, and senescent HSCs (157–159). HSC activation and fibrogenesis are accompanied by a loss of quiescent HSCs and their homeostatic and hepatoprotective functions (28) (Figure 2). Accordingly, CAR T cell–mediated depletion of activated HSCs not only decreased liver fibrosis, but also improved homeostasis in metabolic dysfunction-associated steatohepatitis (160).

### Lung fibrosis.

scRNA-seq and lineage-tracing in mice have shown that PDGFRα^+^ fibroblasts and PDGFRβ^+^ pericytes/adventitial fibroblasts migrate and proliferate at sites of injury, where they turn into activated fibroblasts and myofibroblasts, with both fibroblast activation and proliferation contributing to fibrosis and worsening of lung function (12, 57, 117, 161–166). Among these cells, the alveolar fibroblast represents a major source of activated ECM-producing fibroblasts after alveolar injury, with only minor contributions from pericytes and adventitial fibroblasts (Figure 2), as demonstrated by lineage-tracing and scRNA-seq studies that show inflammatory and fibrotic fibroblasts arising from this cell type (12, 117). *CTHRC1^+^* fibroblasts emerge after lung injury and mark a highly fibrogenic fibroblast population, localized at the leading edge of fibrogenesis (117, 161). These studies also identified novel transcription factors that contribute to fibrogenic activation, such as Runx1 and Runx2 (167, 168). While recent data suggested alveolar fibroblasts as central mediators of lung fibrosis in models of alveolar injury, it is likely that injury to the conducting airways or bronchovascular bundles could entrain fibroblasts in those locations to become inflammatory and/or activated fibroblasts (Figure 2). Fibroblast proliferation represents a key driver of lung fibrosis and contributes to the worsening of lung function, as demonstrated by the genetic ablation or inhibition of proliferating fibroblasts.

### Intestinal fibrosis.

The development of intestinal fibrosis represents a major complication of IBD, such as Crohn’s disease (CD) and ulcerative colitis (UC) (169). While UC is restricted to the mucosa and submucosa, CD involves all layers of the GI wall and can lead to progressive fibrosis and complications such as strictures, requiring surgical resection in a large fraction of patients (169). Consistent with its transmural nature, scRNA-seq studies identified that fibroblasts may originate from the expanded wrapping of mesenteric adipose tissue, also termed creeping fat, in patients with CD (170). This highly activated CTHRC1^+^ fibroblast population contributes to intestinal strictures and is markedly mechanosensitive, characterized by high YAP/TAZ signatures (170). In UC, scRNA-seq studies, besides an expected depletion of epithelial cells and a concurrent enrichment of fibroblasts and inflammatory cells, qualitative changes in fibroblasts were detected (5, 10). While many fibroblast subsets are present in both healthy individuals and patients with UC (5, 10), Smillie et al. described a profound expansion of inflammation-associated fibroblasts, enriched for the profibrogenic cytokine IL11 (171) as well as IL24, and IL13RA2 (10), in patients with IBD. As these inflammation-associated fibroblasts comprise WNT2B^+^ and WNT5B^+^ subsets, they may represent a distinct cellular state along the crypt-villus axis (10). Importantly, inflammation-associated fibroblasts were strongly associated with resistance to anti-TNF therapy, possibly through their expression of the receptor for oncostatin M, a cytokine that drives intestinal inflammation and predicts response to TNF-neutralizing therapy (172). Kinchen et al. identified an expansion of a novel stromal CD24^+^ population in patients with IBD, enriched for proinflammatory genes such as CCL19, CCL21, TNFSF14/LIGHT, CD74 and IL33, suggesting features of inflammatory, antigen-presenting and FRC fibroblasts (5). A similar inflammatory FRC-like fibroblast subtype, expressing high levels of CCL19 and IL33, was increased in DDC-treated mice and may restrict colonic epithelial cell proliferation while inducing the expression of stemness genes, such as Lgr5, via IL6 and TNFSF14 (5). Fistulas in CD contain, in addition to the above-described populations, a distinct population of fistula-associated fibroblasts that may drive fistula formation through epithelialization, remodeling, and immune cell interactions (173). Together, these studies show the dynamic crosstalk between inflammatory, mesenchymal, and epithelial cell compartments and their effect on inflammation and regeneration in IBD and, in case of CD, a role in stricture and fistula formation. An improved understanding of fibroblast functions may allow targeting disease-driving components of this multicellular network.

### Kidney fibrosis.

Tubulointerstitial fibrosis is a chronic and progressive process triggered by kidney injury and aging (24, 129). Kidney fibrosis is involved in virtually all chronic kidney diseases, affecting 10% of the global population (174). scRNA-seq and tracing studies in patients and mice have shown that activated fibroblasts and myofibroblasts can arise from multiple sources (6, 57, 63, 175), with three PDGFRβ^+^ populations as dominant contributors: (a) Notch3^+^RGS5^+^Pdgfra^–^Pdgfrb^+^ pericytes, (b) Meg3^+^Pdgfra^+^Pdgfrb^+^ fibroblasts, and (c) Colec11^+^Cxcl12^+^Pdgfrb^+^ inflammatory fibroblasts (6). These data indicate that the two distinct fibroblast populations serve as major sources of fibrosis, whereas pericytes contribute a smaller part. Earlier studies that have suggested that pericytes are the major source of myofibroblasts in murine kidney fibrosis have used Cre drivers that recombine in both pericytes and fibroblasts (63). Targeting of PDGFRβ^+^ cells via a CAR T cell approach demonstrated not only reduced fibrosis, but also stabilization of kidney function, as estimated by glomerular filtration rate (176). During chronic kidney injury, the conversion of erythropoietin-producing Norn cells to myofibroblasts (48) leads to renal anemia in patients. Together, these studies highlight the central role of fibroblasts in kidney fibrosis and point toward PDGFRβ^+^ cells as targets for the reduction of fibrosis and stabilization of kidney function (Figure 2).

## Fibroblasts in cancer

**Figure 3:**
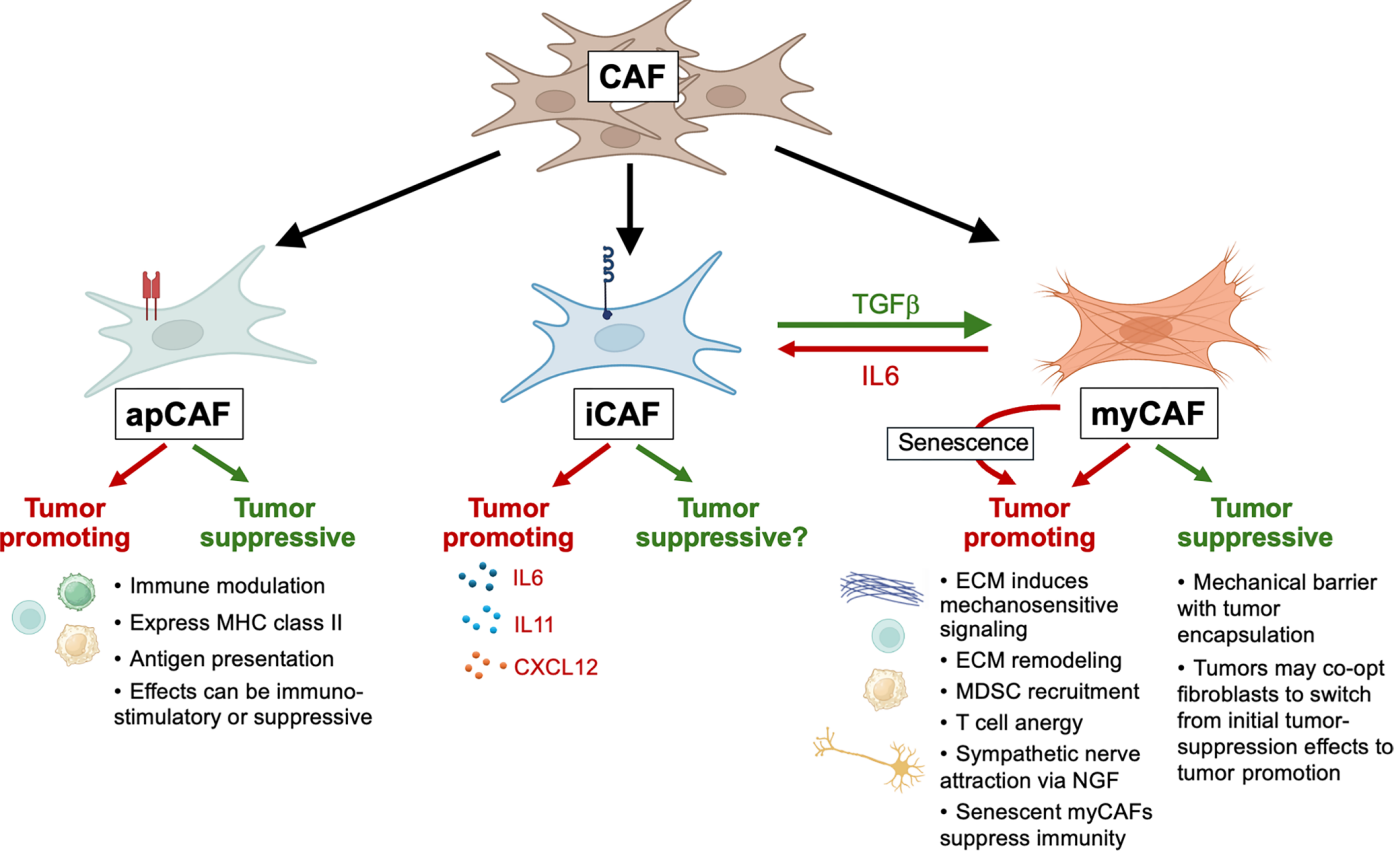
Cancer-associated subpopulations and functions in cancer. Shown are different cancer-associated fibroblast (CAF) states and their functions in cancer, including myofibroblastic CAF (myCAF), inflammatory CAF (iCAF), and antigen-presenting CAF (apCAF).

CAFs are the major cell type comprising the tumor stroma, where they generate ECM and promote cancer progression, immunosuppression, and therapy resistance (Figure 3). Interestingly, some preclinical studies have also reported that fibroblasts, CAFs, and their mediators may also restrain tumors, particularly in the pancreas and liver (11, 56, 114, 177–180) (Figure 3). One hypothesis to explain this apparent paradox is that neoplastic cells may initially be slowed from progression by CAFs until appropriate growth-promoting signals are acquired (181). Alternatively, there may be subsets of CAFs that play distinct roles in tumor progression and tumor suppression. Indeed, similar to fibroblasts in organ fibrosis, CAFs exhibit a high range of diverse subtypes and substates (3, 4, 7, 181, 182).

### CAF diversity.

scRNA-seq–based studies have been essential to resolve three major CAF subtypes, including myofibroblastic CAFs (myCAFs), inflammatory CAFs (iCAFs), and antigen-presenting CAFs (apCAFs) (74, 84). These subtypes occupy partially segregated niches, can interconvert under local cues, and together influence scaffold tissue mechanics, paracrine signaling, angiogenesis, neurogenesis, and immunity (183–186). myCAFs are characterized by high αSMA, contractile cytoskeletal programs, and abundant ECM expression (84, 181, 183), representing terminally differentiated and highly fibrogenic LRRC15^+^ myofibroblasts (3, 4). Functionally, myCAFs are the principal architects of desmoplasia, which may drive tumor growth through stiffness and the activation of mechanosensitive pathways and form a physical and functional barrier for immune cells (84, 181, 183). MyCAFs may be further subclassified by transcriptional methods, revealing a distinct subtype of myCAFs with senescent features that was shown to suppress immunity by inhibiting NK cells in breast cancer and attracting dysfunctional myeloid cells in pancreatic cancer (187, 188). iCAFs are characterized by a cytokine/chemokine-rich secretome in response to IL1/NF-κB and JAK/STAT signaling (84, 181, 183). Through recruitment and polarization of myeloid cells and modulation of T cell states, iCAFs remodel the immune microenvironment while providing trophic support to cancer and endothelial cells (74). Reciprocally, iCAFs can respond to oncostatin M secreted by myeloid cells to promote pancreatic cancer progression and metastasis (189). Notably, TGF-β represents a switch between myCAF and iCAF states, promoting myCAF differentiation and simultaneously inhibiting iCAF differentiation by suppressing IL1 receptor expression (84, 190). apCAFs are reminiscent of antigen-presenting fibroblasts, expressing high levels of MHC class II genes and CD74 but often lacking classical costimulatory molecules (84, 191) and, in some tissues such as the pancreas, they may be derived from mesothelial cells (85). apCAFs are found in clusters near immune aggregates and can present antigen to CD4^+^ T cells and shape local T cell phenotypes. In a recent study of 15 different tissues and tumors, apCAFs were further subdivided into mesothelial-like apCAFs, located near cancer cells, and fibrocyte-like apCAFs in lymphocyte niches (192). Moreover, the ontogeny of CAFs may additionally contribute to diversity, with CAFs arising from the same cellular sources as activated fibroblasts and myofibroblasts in fibrosis (181). For example, in pancreatic cancer, iCAFs have been hypothesized to arise from Pi16^+^ resting fibroblasts, whereas myCAFs have been suggested to arise from Col15a1^+^ and Eng^+^ fibroblasts (71, 193). Additional CAF subtypes continue to emerge, including vascular CAFs, that may be derived from pericytes (181, 194, 195).

### Tumor suppression by fibroblasts.

Tumor suppression by fibroblasts can occur in different stages of carcinogenesis (181, 196). In homeostasis and chronic disease, healthy fibroblasts have been ascribed tumor-suppressive roles (179, 197, 198) (Figure 3). One mechanism underlying these tumor-suppressive effects is the production of homeostatic matrix (56, 179). The exact nature of the tumor-suppressive homeostatic ECM is unknown and may be organ-specific, with collagen XV as one tumor-suppressive basement-associated ECM candidate (56). Another mechanism is contact inhibition, by which healthy fibroblasts suppress the growth of tumor cells (197). Furthermore, homeostatic cytokines secreted by fibroblasts, such as HGF or RSPO3, are important for epithelial health, and their absence has been associated with increased liver cancer (11, 114). CAF may also suppress the growth of already developed tumors in some settings and organs (199–201). CAF-derived type I collagen may contribute to the active encapsulation of tumors by the ECM, leading to restrained tumor growth (199). Type I collagen has also been shown to play a role in immunostimulation in pancreatic cancer, with immunosuppression and accelerated tumor growth after its deletion (200). apCAFs may exert antitumor effects by stimulating antitumor immunity (191). In pancreatic cancer, meflin labels tumor-suppressive CAFs, which may actively contribute to tumor suppression (180).

### Tumor promotion by fibroblasts.

Tumor promotion by fibroblasts can occur in different stages. Fibroblast activation may precede and contribute to cancer development as seen in chronic liver disease (114, 196), in some forms of breast cancer (202), and in the premetastatic niche (203). In established tumors, tumor cells can activate and reprogram fibroblasts through paracrine cues such as TGF-β, IL1, PDGF, and hedgehog (190, 201, 204); contact-dependent signals such as Notch/Jagged (205, 206); exosomes (205); as well as metabolites (207–209). In response, fibroblasts differentiate into CAF subsets that support growth, invasion, and immune evasion through reciprocal tumor-stroma interactions (Figure 3). While CAF-derived collagen XV was found to restrict malignant cell growth, fibroblasts that produce this collagen themselves were shown to support neoplastic cell growth in pancreas cancer, underscoring the complex relationship between CAFs and tumor cells (56, 71). CAF-produced ECM, including fibrillar collagens, fibronectin, and hyaluronan, with further crosslinking by LOX or LOXL, changes the tumor environment with increased stiffness and amplification of ECM-regulated and mechanosensitive pathways, such as integrin/FAK and YAP/TAZ, which promote tumor cell proliferation, stemness, EMT, and invasion (210–212). In parallel, tumor-promoting CAFs secrete growth factors (TGF-β, PDGF, FGF, HGF, EGF receptor ligands), inflammatory cytokines (IL6, LIF), chemokines (notably CXCL12), neurogenesis factors (e.g., NGF), and metabolites (lactate, alanine, glutamine, kynurenine) that sustain tumor cell proliferation and survival and promote metabolic rewiring, angiogenesis, and neurogenesis, while contributing to immune exclusion (186, 209, 213–216). For example, gradients of CXCL12 released by CAFs restrict the entry or positioning of effector T cells, and blocking CXCL12 or its receptor CXCR4 increases intratumoral T cells and improves PD-1 or PD-L1 therapy in preclinical models and early clinical studies (217, 218). Furthermore, ECM can affect both the entry and activation state of immune cells into tumors and often leads to suppressed antitumor immunity (219). An additional key role of myCAFs is their relationship to the high-tension tumor-promoting mechanobiology in cancer, which remains area of active investigation (220, 221). Finally, ADAM12 represents a fibroblast checkpoint, which controls TGF-β–driven myofibroblast activation, T cell responses, and antitumor immunity (222).

### Fibroblast-mediated therapy resistance.

One of the most relevant clinical aspects of CAF biology is the ability of CAFs to promote resistance to a wide range of treatments. CAFs negatively effect on therapeutic responses through different mechanisms, which include stimulating the survival of neoplastic cells through mechanosensitive transcriptional programs, cytokines (IL6, IL17A, HGF, IGF), and vesicle-based transfer of bioactive molecules that may render cells resistant to cell death. Additionally, CAFs may create stromal barriers through impaired perfusion of tumors and the sequestration of chemotherapy by CAFs (223) and reduce drug potency through the promotion of certain cell states, such as a cancer stem cell phenotype that is resistant to many forms of therapy, including chemotherapy and targeted therapies (224–230). Furthermore, CAFs have been associated with resistance to immunotherapy, mediated by an upregulation of immune checkpoints such as PD-L1 and CTLA4 on regulatory T lymphocytes (231), the secretion of IL33 by CAFs, and the immune suppressive features of senescent myCAFs (187, 188, 232). Collectively, these therapeutic resistance characteristics of CAFs provide multiple avenues to mechanistically neutralize these features in combination with other anticancer therapies (230).

## Understanding and targeting fibrosis resolution pathways

Although fibrosis was long considered irreversible, accumulating evidence now indicates that it can be resolved under certain conditions when the underlying disease is cured (233). The three key steps of the resolution process include (a) the degradation of the fibrotic ECM, (b) the elimination of activated fibroblasts and myofibroblasts, and (c) regeneration to restore normal tissue architecture. The intrinsic ability of organs to regenerate may determine the extent of fibrosis resolution achievable. Accordingly, the liver, as a prototypical regenerative organ, shows a high capability to resolve fibrosis and even cirrhosis in mice and patients (22, 234–236). In contrast, the lung and kidney may only have a limited ability to resolve fibrosis, as highlighted by pancreatic transplantation in patients with diabetic nephropathy (233, 237–240). The removal of activated fibroblasts and myofibroblasts is achieved through multiple mechanisms. Apoptosis reduces the number of activated fibroblasts and myofibroblasts by activated CD8^+^ T cells and via Fas-dependent mechanisms in the lung and liver (241–243), whereas reprogramming toward less activated states (244, 245) may restore homeostasis. Accordingly, loss of Fas in fibroblasts prevented the homeostatic resolution of bleomycin-induced lung injury with persistence of profibrotic fibroblasts (243). In addition to reduced ECM synthesis, ECM degradation is critical, as it not only allows for restoring normal tissue architecture but also reduces stiffness and decreases mechanosensitive pathways such as YAP/TAZ, that maintain the identity and survival of activated fibroblasts (246). While fibroblasts themselves show an increased expression of MMPs during fibrosis resolution, it is believed that macrophages remove the bulk of ECM in this setting (22, 247, 248). Long-lasting chronic injury and fibrogenesis lead to a high degree of ECM crosslinking, rendering the ECM more resistant to degradation. Finally, epithelial regeneration and restoration of near-normal tissue architecture are important for organ function. Since fibroblasts have a key role in wound healing and regeneration, it is conceivable that the reprogramming of fibroblasts toward more quiescent and homeostatic states may contribute to the restoration of epithelial homeostasis (11, 28). Examples are the deactivation of HSCs in the liver (244, 245) and the dedifferentiation of lung myofibroblasts after activation of PPARγ (249). Accordingly, CAR T cell–mediated ablation of FAP^+^ HSCs in mice not only reduces liver fibrosis, but also restores homeostasis in metabolic dysfunction–associated steatohepatitis (160). Inhibition of fibrogenic mediators may therefore not only attenuate fibrosis, but also restore homeostasis (28, 175). Finally, macrophage-mediated restoration has entered clinical trials in the liver and may represent a powerful strategy to improve fibrosis and regeneration,and return organs, such as the liver, to homeostatic states (250).

### Conclusion and outlook.

scRNA-seq, combined with functional studies in mice, has provided key insights into the phenotypic and functional diversity of fibroblasts in all major organs. While fibroblast subtypes have adapted to serve many organ-specific functions in homeostasis and disease, there are also many conserved features between organs. Further snRNA-seq–based studies using fibroblasts from different organs from the same donor, as well as developmental barcoding of fibroblast lineage, as done for neural compartmentalization (251), may help to further characterize shared and distinct features as well as fibroblast lineages across organs. By harnessing novel spatial biology approaches of transcriptional and metabolic profiling, insights into how fibroblast subtypes create and maintain tissue niches will be possible. Together, deeper insights into fibroblast biology will enable the development of novel therapies that target pathogenic unique and shared fibroblast populations and functions in wound healing, fibrosis, and cancer. The increasing recognition of the homeostatic functions of fibroblasts may also stimulate the development of novel therapies that bolster epithelial health, regeneration, and function across organs. Understanding key regulators of this fibroblast balance may therefore unlock therapeutic opportunities for the treatment of chronic diseases to reduce the burden of fibrotic sequelae while increasing regeneration and organ function.

## Funding support

This work is the result of NIH funding, in whole or in part, and is subject to the NIH Public Access Policy. Through acceptance of this federal funding, the NIH has been given a right to make the work publicly available in PubMed Central.

- NIH 5R01DK128955, 5R01CA262424, and 5P30DK132710 to RFS and 5P30CA45508, U01CA210240, R01CA229699, U01CA224013, 1R01CA188134, and 1R01CA249002 to DAT.
- German Research Foundation Walter Benjamin grant (project 524949814) to MS.
- American Liver Foundation postdoctoral research fellowship to MS.
- Lustgarten Foundation to DAT, where he is a distinguished scholar and director of the Lustgarten Foundation–designated Laboratory of Pancreatic Cancer Research.
- Donaldson Foundation fellowship to SN.

## Conflict of interest

DS is a founder, is a paid consultant, and owns stock in Pliant Therapeutics; serves on the scientific review board for Genentech and the Inflammation Scientific Advisory Board for Amgen; and is listed as inventor on patents owned by the UCSF describing the uses of antibodies and small molecules targeting integrins and on two pending patents describing methods to engineer cells with synthetic circuits to deliver genetically encoded therapeutics to the lungs and to regions of tissue fibrosis.

DAT is a share holder, scientific cofounder, and scientific advisory board member of Mestag Therapeutics; serves on the scientific advisory boards of Triana Biomedicines and Kayak Therapeutics; and is a scientific editor of the Journal of Experimental Medicine and the chief scientist of the Lustgarten Foundation.

RK is a cofounder and shareholder of Sequantrix GmbH and has received research funding from Travere Therapeutics, Galapagos, Novo Nordisk, Ask Bio, and Chugai.

## Figures and Tables

**Table 1:**
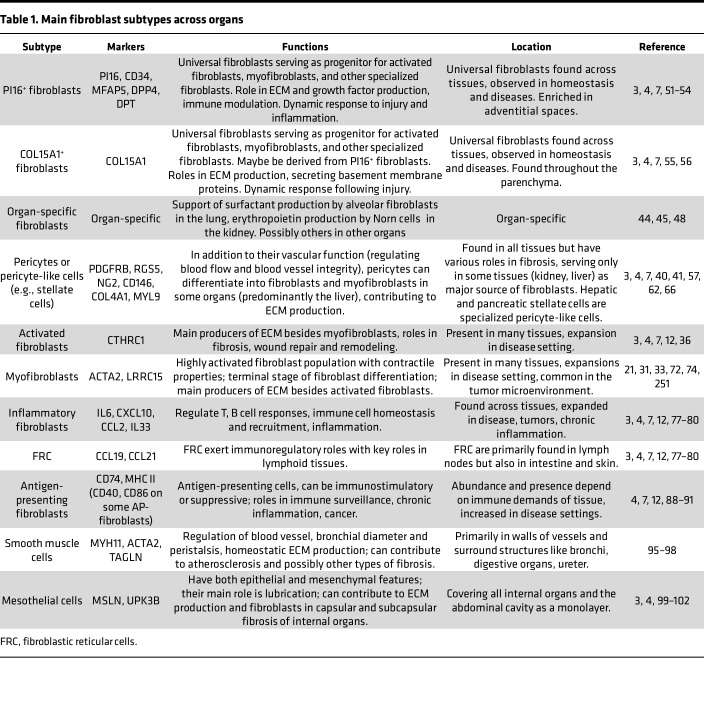
Main fibroblast subtypes across organs

## Version 1 07/08/2026

Electronic publication
